# Knowledge and self-care management practices of asthma among Bangladeshi women

**DOI:** 10.1016/j.aprim.2025.103274

**Published:** 2025-04-10

**Authors:** Mahamaya Acharjee, Md. Monir Hossain Shimul, Salamat Khandker

**Affiliations:** Department of Public Health, Daffodil International University, Dhaka, Bangladesh

Asthma is a major global health concern, affecting over 300 million individuals worldwide, with a disproportionately high burden in low- and middle-income countries like Bangladesh. It is characterized by recurrent episodes of wheezing, breathlessness, and airway inflammation, significantly impairing the quality of life of affected individuals. In Bangladesh, asthma accounts for 1.24% of annual deaths, and among women, socio-economic and cultural constraints further exacerbate self-care challenges. Despite the availability of treatment, self-care practices remain inconsistent, often due to limited awareness, financial barriers, and inadequate access to healthcare facilities. Studies suggest that knowledge about asthma management positively correlates with improved adherence to treatment regimens, but evidence on asthma self-care management among Bangladeshi women remains scarce. Addressing this knowledge gap is crucial to developing targeted interventions to improve asthma outcomes in this vulnerable population.^1^, ^2^

A cross-sectional study was conducted at the National Asthma Center (NAC) in Dhaka between December 2023 and March 2024, involving 180 adult female asthma patients. A purposive sampling technique was employed, and structured, interviewer-administered questionnaires were used to assess participants’ knowledge, attitudes, and self-care management practices. The questionnaire included key domains such as symptom recognition, trigger avoidance, medication adherence, and healthcare-seeking behavior. Participants’ responses were scored and categorized into different levels of knowledge and practice. Descriptive and inferential statistical analyses were performed using SPSS, with Chi-square and Pearson correlation tests applied to assess associations between knowledge and self-care practices.

The results revealed that 61.1% of participants demonstrated good knowledge about asthma, with most recognizing key symptoms such as breathlessness (97.8%), wheezing (92.2%), and coughing (95.0%). However, only 38.9% exhibited good self-care practices, highlighting a significant gap between awareness and implementation. While 100% of respondents acknowledged the importance of avoiding smoking, 27.8% reported irregular medical consultations due to financial constraints (3.9%) and distance to healthcare facilities (11.2%). The study found a statistically significant positive correlation (*p* < 0.05) between knowledge levels and self-care practices, indicating that higher awareness contributes to improved asthma management (Table 1). Despite this, many patients reported difficulties in adhering to prescribed medication regimens, with affordability and accessibility cited as major concerns. The reliance on traditional healing practices and misinformation about inhaler use also emerged as barriers to effective asthma control. The study further revealed that women with higher educational attainment were more likely to engage in proactive self-care practices, suggesting that literacy plays a crucial role in health behavior.^3^, ^4^

**Table 1:** Association between level of knowledge with level of practice.

Knowledge	Practice					Total	*χ*^2^	*p*
	Poor	Average	Satisfactory	Good	Excellent			
Satisfactory	2(3.84%)	10(19.23%)	25(48.07%)	14(26.92%)	1(1.92%)	52(100.0)	75.667	0.001
Good	0(0.0%)	18(16.36%)	30(27.27%)	50(45.45%)	12(10.92%)	110(100.0)		
Excellent	0(0.0%)	0(0.0%)	4(22.22%)	6(33.33%)	8(44.44%)	18(100.0)		
Total	2(1.11%)	28(15.56%)	59(33.1%)	70(38.89%)	21(11.67%)	180(100.0)		

These findings underscore the urgent need for targeted interventions to bridge the gap between asthma knowledge and self-care practices. Previous research has shown that comprehensive asthma education programs significantly improve medication adherence and reduce emergency hospital visits. However, in resource-constrained settings like Bangladesh, structural barriers such as inadequate healthcare infrastructure and high out-of-pocket costs impede effective disease management. Community-based awareness campaigns, mobile health initiatives, and integrating asthma education into routine clinical care are potential strategies to enhance self-care practices among affected women. Additionally, policy interventions aimed at subsidizing asthma medications and expanding healthcare services in rural and peri-urban areas are critical to ensuring sustainable improvements in disease outcomes. The study's findings align with global research, which emphasizes the necessity of patient-centered approaches in asthma management.^5^, ^6^

In conclusion, while Bangladeshi women with asthma exhibit moderate levels of knowledge, their self-care practices remain suboptimal due to systemic and socio-economic barriers. A multifaceted approach, combining education, healthcare accessibility improvements, and policy support, is essential to improving asthma self-management in this population. Future research should focus on large-scale, multi-center studies to validate these findings and develop evidence-based strategies for integrating asthma care into primary healthcare services. Strengthening health literacy, reducing financial constraints, and leveraging technology-driven solutions will be instrumental in mitigating the burden of asthma in Bangladesh.

## CRediT authorship contribution statement

1. Mahamaya Acharjee: Conceptualization, data curation, formal analysis, investigation, methodology, writing – original draft.
2. Md. Monir Hossain Shimul: Methodology, formal analysis, visualization, writing – original draft, and writing – review & editing.
3. Salamat Khandker: Methodology, supervision, writing – review and editing.

## Ethical considerations

The study was conducted in compliance with ethical guidelines, with approval obtained from the Research Ethics Committee of the Faculty of Health and Life Sciences, Daffodil International University (Approval Reference: FAHSREC/DIU/2023/SMIG-36). Written consent has been obtained from all participants.

## Funding

This research did not receive any specific grant from funding agencies in the public, commercial, or not-for-profit sectors.

## Conflict of interest

The authors and corresponding authors declare no competing interests.
